# The Role of Biomaterials as Angiogenic Modulators of Spinal Cord Injury: Mimetics of the Spinal Cord, Cell and Angiogenic Factor Delivery Agents

**DOI:** 10.3389/fphar.2018.00164

**Published:** 2018-02-27

**Authors:** Luís A. Rocha, Rui A. Sousa, David. A. Learmonth, António J. Salgado

**Affiliations:** ^1^Life and Health Sciences Research Institute (ICVS), School of Medicine, University of Minho, Braga, Portugal; ^2^ICVS/3B’s – PT Government Associate Laboratory, Braga, Portugal; ^3^Stemmatters, Biotecnologia e Medicina Regenerativa SA, Barco, Portugal

**Keywords:** spinal cord injury, angiogenesis, vascularization, biomaterials, growth factors

## Abstract

Spinal cord injury (SCI) represents an extremely debilitating condition for which no efficacious treatment is available. One of the main contributors to the inhospitable environment found in SCI is the vascular disruption that happens at the moment of injury that compromises the blood-spinal cord barrier (BSCB) and triggers a cascade of events that includes infiltration of inflammatory cells, ischemia and intraparenchymal hemorrhage. Due to the unsatisfactory nature of revascularization following SCI, restoring vascular perfusion and the BSCB seems an interesting way of modulating the lesion environment into a regenerative phenotype, with a potential increase in functional recovery. Certain biomaterials possess interesting features to enhance SCI therapies, and in fact have been applied as angiogenic promoters in other pathologies. The present mini-review intends to highlight the contribution that biomaterials could make in the development of novel therapeutic solutions able to restore proper vascularization and the BSCB.

## Introduction

Vascular disruption following SCI plays a critical role in triggering some of the secondary events associated with this injury such as uncontrolled infiltration of inflammatory cells and ischemia. The extension of intraparenchymal hemorrhage that appears as a consequence of SCI has been correlated with the area occupied by the cystic cavity (Noble and Wrathall, 1989a,b). Two days following the incidence of SCI, the density of blood vessels decreases and only residual levels are observed at the injury site (Ng et al., 2011). Angiogenesis (the growth of blood vessels from preexisting ones) initiates 3 to 4 days after injury and is seen up to 1 week after SCI (Casella et al., 2002; Dray et al., 2009). Although different studies showed revascularization similar to pre-lesion levels or even fivefold higher, these new vessels are not associated with astrocytes, pericytes, or neurons. Moreover, no restoration of Glut-1 transporters (essential for the continuous supply of glucose to metabolically unbalanced neurons) was seen until 2 weeks following SCI (Ng et al., 2011). This demonstrates that endothelial cells (ECs) within newly formed vessels are not in the desired phenotype, and hence, do not guarantee satisfactory recovery from the initial disruption. Different therapeutic approaches have correlated improvements in functional recovery with augmented densities of blood vessels in spinal cord tissue (Glaser et al., 2006; Kaneko et al., 2007). This is further sustained by the elevated metabolic need of neurons, making these cells more susceptible to damage during prolonged ischemia (Attwell and Laughlin, 2001). Moreover, the proper restoration of the blood-spinal cord barrier (BSCB) may help control the influx of inflammatory cells into the damaged spinal cord and direct the inflammatory response toward a regenerative path.

The angiogenic response following SCI appears to be modulated by a complex interplay between different proteins (Kundi et al., 2013). Among these, vascular endothelial growth factor (VEGF) is thought to possess an ambiguous role in SCI angiogenesis, since some studies show that it has no impact (Benton et al., 2009) or increases the lesion volume (Benton and Whittemore, 2003). Others correlate the administration of VEGF with higher densities of blood vessels, tissue sparing and improved motor outcomes (Widenfalk et al., 2003; Kim et al., 2009). This might be due to the roles that different VEGF isoforms have on SCI angiogenesis and pathophysiology, the route of administration, dose and short circulatory half-life (Crafts et al., 2015). The synergistic action between VEGF and angiopoietin-1 (ANG-1) in the context of SCI was explored by administering these GFs into the lesion immediately after injury. This therapeutic approach promoted vascular stabilization, reduced the lesion volume and functionally translated into improved locomotor behavior in the chronic phase (Herrera et al., 2010). ANG-1 limits vascular permeability, helping to maintain the integrity of the BSCB by reducing the number and size of the gap junctions between ECs (Baffert et al., 2006). Additionally, ANG-1 contributes to the stabilization and maturation of blood vessels during the final phases of angiogenesis (Wong et al., 1997). During an SCI event, mRNA expression and protein levels of ANG-1 are diminished, contributing to decreased integrity of the BSCB (Durham-Lee et al., 2012). Delivery of ANG-1 into an SCI animal model resulted in the preservation of blood vessels at the injury site whilst improving locomotor function and permanently rescuing white matter, features that correlated with increased perfused blood vessels (Han et al., 2010). Matrix metalloproteinases (MMPs) have also been implicated in the vascular events following SCI (Verslegers et al., 2013). This class of enzymes are capable of cleaving all the extracellular matrix (ECM) components and are key to cell migration (Nagase et al., 2006). Depletion of MMP-2 in a contusion SCI animal model led to a decrease in EC division during the first 2 weeks following SCI and to significant vascular decline 21 days post-injury (Trivedi et al., 2016). On the other hand, up-regulation of MMP-11 during the acute phase of SCI seemed to be involved in disrupting the BSCB and increasing its permeability. The expression of MMP-11 reaches a maximum at 24 h after injury, followed by a dramatic reduction at 72 h and then undetectable at 7 days (Noble et al., 2002). These results open the possibility of using modulation of the expression of MMPs as a target for improved vascularization and functional outcomes in SCI.

Biomaterials can aid modulation of the vascular response following SCI via two distinct mechanisms, namely acting as vehicles for the delivery of pro-angiogenic molecules (Yu et al., 2016) or as ECM-mimetic platforms that support cell growth and proliferation (Rauch et al., 2009). The capacity that biomaterials have for protecting cells and therapeutic agents from the harsh conditions found in SCI lesion sites puts them in a privileged position for the development of targeted regenerative therapies. Furthermore, this is complemented by the possibility of tailoring their mechanical properties to match native ECM and to their biocompatible and biodegradable characteristics (Haggerty et al., 2017). Biocompatibility reduces the risk of triggering toxic or immunological responses within the CNS, a feature that if not fulfilled could induce chronic inflammation at the biomaterial interface resulting in the restrain of the scaffold by an avascular glial scar (Orive et al., 2009; Slaughter et al., 2009; Sensharma et al., 2017). The natural degradation processes of biomaterials under physiological conditions, without originating toxic metabolites, represents another advantage in SCI as it eliminates the need of follow-up surgical procedures for their subsequent removal. Tuning the degradation of these materials allows control of the rate of release of angiogenic factors thereby enabling the optimization of bioavailability and therapeutic concentration (Sensharma et al., 2017). Therefore, the present mini-review intends to give an overview of the potential of biomaterials as modulators of vascularization in SCI lesion sites. Emphasis will be given to their capacity to deliver neurovascular agents in a localized manner and to their suitability to act as ECM-like structures that aim to restore vascularization and BSCB following SCI.

## Biomaterials as Tools for the Modulation of Angiogenesis and Vascularization

### ECM-Like Platforms to Support Angiogenesis and Vascularization

In their native environment, cells are embedded in a three-dimensional ECM responsible for providing adequate mechanical and physical cues that provide instructions to engage in specific behaviors (Guvendiren and Burdick, 2013). Additionally, the ECM confers to cells mechanical support and protection from the external environment (Slaughter et al., 2009). This structure interacts with angiogenic GFs, to coordinate their bioavailability, concentration, and signaling (Martino et al., 2015). For instance, VEGF disseminates across the interstitial space and binds both to the ECM and receptors on the surface of cells creating a concentration gradient that attracts endothelial sprouts in the direction of hypoxic regions (Vempati et al., 2011). Cell-derived proteases regulate the availability of functional GFs linked to the matrix through their capacity to degrade ECM constituents or by cleaving these molecules into isoforms with reduced bioactivity that are incapable of binding to the ECM (Briquez et al., 2016).

Given the importance of the ECM during angiogenesis, developing precise analogs of this structure to therapies that aim to restore vascular perfusion seems particularly promising. On this front, biomaterials seem a perfect fit due to their ability to mimick the mechanical properties of the ECM and to provide specific molecular cues (Devolder and Kong, 2012). Commonly, these biomaterials can be of natural origin (ECM-derived or otherwise) or synthetic. Hydrogels from ECM-derived proteins like fibrin, collagen or gelatin are normally used and can be modified regarding their mechanical properties, degradability, cell adhesion, and GF-bearing capacity to a limited extent (Browne and Pandit, 2017). Natural non-protein biomaterials, including alginate (Dalheim et al., 2016), pectin (Neves et al., 2015), dextran (Riahi et al., 2017) and gellan gum (Gomes et al., 2016), are bioinert and require functionalization with appropriate adhesion motifs to acquire biological activity. Additionally, mechanical properties and degradation profiles are adjustable by varying the degree and nature of crosslinking or by including cell degradable peptides, respectively (Lau and Wang, 2013). On the other hand, synthetic biomaterials such as polyethylene glycol (PEG), poly(ε-caprolactone) (PCL) and poly(lactic-co-glycolide) (PLGA) are excellent alternatives to natural polymers due to the possibility to modulate their properties to a greater extent. Moreover, they can be obtained in a reproducible manner, which enables control over molecular weight, mechanical strength, degradation, crosslinking degree, and cell adhesive behavior (Zhu and Marchant, 2011). Therefore, incorporating cell adhesion motifs together with protease-sensitive sites represents a common strategy to induce angiogenesis and vascularization of natural and synthetic materials and the biomaterial-tissue interface (Hanjaya-Putra et al., 2012; Tsurkan et al., 2013; Chwalek et al., 2015; Jha et al., 2016). Interestingly, by controlling the spatial distribution and density of these molecular cues it is possible to modulate not only the maturation and formation of newborn blood vessels but also the rate at which they degrade the engineered ECM and infiltrate into host tissue or vice versa (Hanjaya-Putra et al., 2011, 2012). Thus, these types of materials can be considered blank canvasses to create tunable platforms that can modulate the angiogenic response in a specific way unlike ECM-derived materials.

### Enhancers of the Delivery of Angiogenic GFs

Delivery of angiogenic GFs has been acknowledged as a promising tool to stimulate angiogenesis and restore vascular perfusion. Nevertheless, clinical translation has proven difficult as these molecules have short *in vivo* half-lives, dosages are sub-optimal and poor retention kinetics (Browne and Pandit, 2017).

Biomaterials provide a route to circumvent some of these problems as they can protect GFs from degradation and can be tuned to release them in a controllable way (Abdeen and Saha, 2017). Consequently, biomaterials can be designed to create a chemical gradient during the release of GFs, mimicking *in vivo* angiogenesis, and affecting the rate of EC invasion, its direction, structure and network formation (Guo et al., 2012; Akar et al., 2015). Biomaterials can be functionalized with more than one type of GFs and further replicate native angiogenesis, a process that depends on distinct concentration gradients and bioavailability of these molecules (Richardson et al., 2001; Shin et al., 2011; Assal et al., 2013; Rufaihah et al., 2017). Indeed, both synthetic and natural biomaterials have been used either by physically entrapping the GFs or by establishing chemical bonds with the matrix (Wang et al., 2009; Anderson et al., 2011; Des Rieux et al., 2014; Mittermayr et al., 2016). Perhaps the best approach to enhance the angiogenic response would be to combine the delivery of GFs with molecules capable of inducing their expression, such as sonic hedgehog (Shh). Consequently, Shh induces the expression of VEGF, Ang-1 and Ang-2, increasing their concentration and leading to the formation of more functional and stable vessels *in vivo* (Pola et al., 2001; Rivron et al., 2012). This methodology enables cells to regulate the secretion of GFs, whilst helping the formation of microgradients and granting the possibility of expressing different GFs simultaneously (Baiguera and Ribatti, 2013).

### Integration of Biomaterials in SCI Angiogenic Therapies

Reestablishing the BSCB and potentiating the recovery of adequate blood supply in SCI would appear a fundamental requirement for efficacious therapies. Han et al. (2010) administered intravenous injections of Ang-1 and C16 (an angiogenic peptide) in a thoracic SCI mouse model and observed neuroprotective action of this treatment materialized by sparing epicenter blood vessels and white matter, increased angiogenesis and reduction of harmful inflammation. Most importantly, these histological findings correlated with significant motor recovery of the animals. Ang-1 reduced vascular permeability, monocyte transmigration as well as microglia/macrophages activation and infiltration (important players in white matter damage). Adding to its effect on preserving blood vessels at injury site, C16 showed pro-angiogenic activity and, noteworthy, also anti-inflammatory properties as it decreased monocyte transmigration across an EC layer *in vitro* (Han et al., 2010). This study clearly demonstrates the potential of developing strategies aiming to restore vascularization following SCI. As depicted in the previous sections, biomaterials can provide interesting platforms to enhance these particular therapies and in fact have shown the capacity to modulate angiogenesis and vascularization following SCI (Bakshi et al., 2004; Rauch et al., 2009; King et al., 2010; Hurtado et al., 2011; Zeng et al., 2011; López-Dolado et al., 2016; Chedly et al., 2017). Accordingly, Duan et al. (2015) utilized neurothrophin-3 (NT-3) loaded chitosan tubes to fill the void left by the transection of rat spinal cords and found that this material promoted nerve growth, neurogenesis and functional recovery of the animals. Thus, this study found an upregulation on genes related to vascular development, angiogenesis and hypoxia response in the NT-3 treatment group, when compared to uninjured and untreated animals (Duan et al., 2015). Differently, Rauch et al. (2009) created a co-culture system consisting of ECs and neural progenitor cells (NPCs) in a biodegradable PLGA scaffold and tested its ability to form functional vessels in an SCI hemisection model. After implantation, this system created a suitable environment for vessel inosculation and angiogenesis in the experimental group, contributing to a 3.5-(PLGA implantation without cells group) and 5-fold (lesioned animals group) increase in number of functional vessels at injury epicenter at 8 weeks. The crosstalk between ECs and NPCs was fundamental due to the secretion of NO by NPCs, which induces the production of VEGF and brain-derived neurotrophic factor on ECs and contribute to further enhance NO production, promoting vessel formation and stabilization. Notably, the co-culture platform seemed to promote the re-establishment of BSCB since half of the vessels in the experimental group were positive to endothelial barrier antigen (a major marker for BSCB). In contrast, all the other cohorts (untreated, PLGA implantation, PLGA and ECs implantation and PLGA harboring NPCs group) had no expression of this marker (Rauch et al., 2009). Even though the authors did not assess BSCB functionality and observed limited regeneration, this work underlines the potential of integrating biomaterial-based ECs transplantation into SCI experimental treatments due to their capacity of reestablishing perfusion and BSCB, helping to modulate a regenerative phenotype. In an interesting approach, López-Dolado et al. (2016) evaluated the regenerative capacity of graphene oxide scaffolds in a hemisection rat model due to its capacity of inducing neuronal and astrocytic growth and neurogenesis. Upon implantation, these scaffolds promoted angiogenesis inside their structure, showing abundant and functional new vessels in their proximity in comparison to lesioned animals without scaffold implantation. Additionally, the scaffolds also seemed to have immunomodulatory capacity due to an increased presence of pro-regenerative macrophages on its interface. On the other hand, infiltration of neurons into the scaffolds was very low and no measurements on functional outcomes were assessed (López-Dolado et al., 2016). Nevertheless, this study presents some encouraging results and it is worth underlining the outstanding conductible properties of graphene, a feature that can play a pivotal role in therapies that apply electric stimulation to induce neural growth (Li et al., 2013; Akhavan et al., 2016). Ropper et al. (2017) implanted a PLGA scaffold encapsulating human mesenchymal stem cells (hMSCs) in a thoracic hemisection rat model to study the potential of this system in SCI recovery. This treatment induced significant moto-sensory improvements regarding untreated animals or the groups where either scaffold insertion or hMSCs transplantation occurred. Additionally, treatment with hMSCs encapsulated in PLGA lead to significant decreases in lesion volume and improvements in neuropathic pain in comparison to controls. Furthermore, histological analysis of spinal cord sections showed an increased angiogenesis around the epicenter (observable by a significant increase in laminin concentration on the treatment group) which together with neurotrophic, anti-inflammatory and neurogenic mechanisms helps explaining the obtained moto-sensory improvements of this experimental approach. Nevertheless, therapeutic differences between the direct application of hMSCs and their prior encapsulation in PLGA may reside in the protective action of the polymer toward the inhospitable environment found on SCI, which was transduced in augmented hMSCs survival upon implantation for that group (Ropper et al., 2017). The positive impact of MSCs on the angiogenesis and vascularization of SCI was probably driven by the secretome of these cells which is extremely rich in pro-angiogenic GFs (Ranganath et al., 2012). Accordingly, several researchers have taken advantage of the aforementioned features of biomaterials to explore delivery of angiogenic GFs in SCI animal models and assess their impact on recovery following injury (De Laporte et al., 2011; Kang et al., 2012; Des Rieux et al., 2014; Wen et al., 2016; Yu et al., 2016). Combinatorial approaches utilizing different angiogenic GFs perhaps represent the best way of attaining better functional outcomes following SCI. Consequently, Yu et al. (2016) delivered PLGA microspheres containing VEGF, ANG-1 and basic fibroblast growth factor into the injury site of a contusion rat model and observed increased axonal growth on the treated animals in comparison to animals that received the empty microspheres. The authors associated these results with increased density of functional vessels and neural precursors recruitment to the injury site. Moreover, these cells closely associated with blood vessels opening the possibility of the microvascular network having a role on axonal guidance and growth across the lesion cavity. This study also found increased expression levels of miR-210 in treated animals, an inducer of VEGF expression, and suppression of ephrin-A3 (negative modulator of neurogenesis), a finding that demonstrates that increased neurogenesis found on the treated group was probably directly due to the GFs administration, broadening their spectrum of action (Yu et al., 2016).

## Conclusion

The application of biomaterials to influence angiogenesis/vascularization in SCI has shown interesting results. Biomaterials can deliver angiogenic GFs efficiently, enhancing their action and replicating some of the features found in native angiogenesis. Moreover, biomaterials protect cells from the harsh environment found in SCI, allowing their proliferation and exertion of biological effects, and a route (in some cases) to bridge the cavity that forms following SCI, allowing neuronal recovery. Addressing SCI vascularization using biomaterials certainly has potential and their incorporation in future therapies may be essential. Indeed due to the complex nature of SCI, unidimensional approaches are unlikely to be the best strategy or succeed. Therefore, integrating revascularization approaches in therapies that provide a means for neuronal growth and to modulate the environment into regenerative pathways is probably the best option moving forward. Due to their versatility, biomaterials can provide excellent platforms to be integrated into the development of more effective therapies.

## Author Contributions

LR drafted the manuscript. RS and DL helped to draft the manuscript and revised it critically. AS conceived the analysis, participated in its design and coordination, helped to draft the manuscript and gave the final approval of the version to be published. All authors read and approved the final manuscript.

## Conflict of Interest Statement

The authors declare that the research was conducted in the absence of any commercial or financial relationships that could be construed as a potential conflict of interest.
